# Genetic characterization of *Salmonella* Infantis from South Africa, 2004–2016

**DOI:** 10.1099/acmi.0.000371

**Published:** 2022-07-05

**Authors:** Jennifer Mattock, Anthony M. Smith, Karen H. Keddy, Emma J. Manners, Sanelisiwe T. Duze, Shannon Smouse, Nomsa Tau, David Baker, Marie Anne Chattaway, Alison E. Mather, John Wain, Gemma C. Langridge

**Affiliations:** ^1^​ Norwich Medical School, University of East Anglia, Norwich, UK; ^2^​ Centre for Enteric Diseases, National Institute for Communicable Diseases, Johannesburg, South Africa; ^3^​ Independant Consultant, Johannesburg, South Africa; ^4^​ Faculty of Health Sciences, University of the Witwatersrand, Johannesburg, South Africa; ^5^​ Microbes in the Food Chain, Quadram Institute Bioscience, Norwich, UK; ^6^​ Gastrointestinal Bacteriology Reference Unit, United Kingdom Health Security Agency, London, UK; ^7^​ Faculty of Medicine and Health Sciences, University of East Anglia, Norwich, UK; ^†^​ Present address: The Roslin Institute, University of Edinburgh, UK; ^‡^​ Present address: European Molecular Biology Laboratory, European Bioinformatics Institute, UK

**Keywords:** *Salmonella *Infantis, eBG31, eBG297, AMR

## Abstract

*
Salmonella* Infantis is presenting an increasing risk to public health. Of particular concern are the reports of pESI, a multidrug resistance (MDR) encoding megaplasmid, in isolates from multiple countries, but little is known about its presence or diversity in South Africa. Whole genome sequences of 387 *S*. Infantis isolates from South Africa (2004–2020) were analysed for genetic phylogeny, recombination frequency, antimicrobial resistance (AMR) determinants, plasmid presence and overall gene content. The population structure of South African *S*. Infantis was substantially different to *S*. Infantis reported elsewhere; only two thirds of isolates belonged to eBG31, while the remainder were identified as eBG297, a much rarer group globally. Significantly higher levels of recombination were observed in the eBG297 isolates, which was associated with the presence of prophages. The majority of isolates were putatively susceptible to antimicrobials (335/387) and lacked any plasmids (311/387); the megaplasmid pESI was present in just one isolate. A larger proportion of eBG31 isolates, 19% (49/263), contained at least one AMR determinant, compared to eBG297 at 2% (3/124). Comparison of the pan-genomes of isolates from either eBG identified 943 genes significantly associated with eBG, with 43 found exclusively in eBG31 isolates and 34 in eBG297 isolates. This, along with the single nucleotide polymorphism distance and difference in resistance profiles, suggests that eBG31 and eBG297 isolates occupy different niches within South Africa. If antibiotic-resistant *S*. Infantis emerges in South Africa, probably through the spread of the pESI plasmid, treatment of this infection would be compromised.

## Data Summary

The Illumina FASTQ files produced in this project are available in the European Nucleotide Archive (ENA) study PRJEB49327. The run accession for each sample is available in Table S1(available in the online version of this article). The assemblies used as the eBG31 and eBG297 references can be found in the ENA study PRJEB49327 with accessions GCA_922393895.1 and GCA_922393915.1 respectively.

## Introduction

Globally, non-typhoidal *Salmonella enterica* (NTS) are estimated to cause 93.8 million cases of gastroenteritis each year, resulting in 155,000 deaths [1]. The majority of patients present with gastrointestinal symptoms but cases can progress to invasive disease [2], presenting as febrile illness [3, 4] and requiring antibiotic therapy [5] including fluoroquinolones, third-generation cephalosporins such as extended-spectrum β-lactams, and trimethoprim-sulfamethoxazole or ampicillin [6–10]. A threat from NTS infection is antimicrobial resistance (AMR); in 2016, fluoroquinolone-resistant *Salmonella* was added to the World Health Organization’s high-priority tier of bacteria with AMR requiring research, exemplifying the risk to public health [11]. Multidrug resistance (MDR), resistance to three or more antibiotic classes, is a public health concern as NTS with MDR are associated with an increased infection severity [12].

High levels of MDR in low- and middle-income countries (LMICs), defined using the World Bank gross national income [13], where there are large numbers of immunocompromised individuals is of particular concern [14]. In South Africa, human immunodeficiency virus (HIV) infection has been associated with invasive salmonellosis [15]. In comparison to England and Wales where HIV infection is at a low level (2016: 0.16%) [16], in South Africa, HIV infection is high (2018: 13.06 %) [17]. High endemic levels of HIV lead to the increased use of antimicrobials which, in turn, leads to an increase in AMR [18].

*S. enterica* serovar Infantis (*S*. Infantis) is becoming an increasingly prevelant serovar; between 2013 and 2020, *S*. Infantis was the fourth most common serovar of *Salmonella* reportedly causing human infection in EU member states [19–25]. In South Africa, *S*. Infantis has been reported as being amongst the the top four serovars causing human infection [26, 27]. Higher frequencies of *S*. Infantis have been reported in Israel, where it accounted for 30% of human cases between 2008 and 2015 [28]. Importantly, *S*. Infantis is the serovar most frequently identified in domestic fowl in EU member states [25]. Conversely, between 2012 and 2014 in South Africa, *S*. Infantis was the 11th most common serovar isolated from farm animals [29].

The population structure of *S*. Infantis, when multilocus sequence typed and clustered with sequence type (STs) that differed by one allele into eBurst Groups (eBGs), consists of two eBGs, eBG31, the most predominant described globally to date, and eBG297 [30, 31]. Similar to other *Salmonella* serovars, the levels of AMR in *S*. Infantis fluctuate globally [32–34], although levels of *S*. Infantis MDR appear higher than *S*. Enteritidis in Turkey and Iran [34, 35]. In Europe, *S*. Infantis has become a noteworthy contributor to MDR in *Salmonella* [7, 36]. In 2016, 31% of isolates from broilers and 70% from broiler meat had an MDR phenotype [37]. Furthermore, in 2014 a unique megaplasmid, plasmid of emerging *S*. Infantis (pESI) that confers MDR, was identified in *S*. Infantis isolates in Israel [38]. Since then pESI-like plasmids have also been identified in *S*. Infantis from Italy, Germany, the Netherlands, Poland, Ukraine, Switzerland, Luxembourg, Romania, Hungary, Denmark, Peru, Turkey, the UK and the USA, suggesting that pESI presence in certain *S*. Infantis strains is advantageous to the pathogen [7, 8, 30, 39–44].

Despite the high levels of AMR reported globally in *S*. Infantis and the purported increased antimicrobial requirements in LMICs with an increased proportion of immunocompromised people [17], little has been reported about African *S*. Infantis. To explore our hypothesis that AMR in *S*. Infantis is a threat in Africa, we analysed *S*. Infantis strains isolated from South Africa, between 2004 and 2016. To investigate whether the population structure of South African *S*. Infantis is similar to that observed elsewhere and the public health concern this pathogen presents, we evaluated the phylogenetic structure, recombination frequency, genetic antimicrobial resistance determinants, plasmid presence and overall gene content in *S*. Infantis.

## Methods

### Isolate selection

Clinical *S*. Infantis strains from the National Institute for Communicable Diseases (NICD) culture collection in South Africa were selected for sequencing to represent all sources tested. These were available with metadata from 2004 to 2016; equal proportions of isolates from each year were included. The NICD participates in routine national laboratory-based surveillance for *Salmonella *, where as part of the Group for Enteric, Respiratory and Meningeal disease Surveillance in South Africa (GERMS-SA) Network, all clinical *Salmonella* isolates (invasive and non-invasive) are collected by voluntary submissions from >200 clinical microbiology laboratories across the country. *Salmonella* isolates from the current NICD culture collection are representative of all clinical isolates (invasive and non-invasive) from across South Africa, but the collection presented here is biased towards invasive isolates as historically it was predominantly blood isolates that were submitted. In total, 395 *S*. Infantis clinical isolates were sequenced and met quality criteria.

To expand the collection where possible, sequenced clinical isolates referred to Public Health England between 2 May 2012 and 15 May 2020 were screened for travel history to South Africa, resulting in one *S*. Infantis strain (SRR1645903) being selected and downloaded from the NCBI BioProject PRJNA248792. The minimal spanning tree of *Salmonella* multilocus sequence typing (MLST) data in Achtman *et al*. was used to identify the closest eBG to eBG31, eBG8 (*S*. Muenchen), to use as an outgroup [31]. The publicly available global database EnteroBase was screened for *S*. Infantis and eBG8 isolates uploaded with the country being South Africa on 4 November 2020 [45]; one eBG31 (ERR6753008) and four eBG8 isolates were included. Isolates from rectal swabs were classed as from stools and isolates from ‘wound swab’ and ‘swab superficial’ were grouped as ‘Other’ (Table S1a).

In total, the entire collection comprised 387 *S*. Infantis sequences, isolated from humans between 2004 and 2020 (Table S1a). Significant differences between the proportion of eBG31 and eBG297 isolates that were invasive were determined using the two population proportions z test calculator [46]. The number of eBG31 and eBG297 isolates on Enterobase, with isolation source niche of human and excluding isolates from South Africa, was counted on 2 February 2022 to enable the comparison between the proportion belonging to either eBG elsewhere.

### Whole genome sequencing

DNA from the NICD isolates from 2004 to 2016 was extracted using the QIAamp DNA Mini Kit (Qiagen). Illumina Nextera XT libraries were prepared for the first 99 samples following the manufacturer’s instructions and paired-end sequenced using a mid-output flow cell on an Illumina NextSeq 500 [47]. Base calling was performed using bcl2fastq on the Cloud Infrastructure for Microbial Bioinformatics [48, 49]. The remaining isolates were prepared as described in Rasheed *et al*. and sequenced on an Illumina NextSeq 500, using BaseSpace (Illumina) to base call [50].

Raw sequence reads were quality checked with FastQC and trimmed using Trimmomatic v.0.36 [51, 52]. MLST was performed using Metric-Oriented Sequence Typer (MOST) (v.1.0) against a validated United Kingdom Health Security Agency (UKHSA) in-house *Salmonella* database and Tablet to assess the quality of borderline sequence typing [53–55]. Sequences were excluded if the MLST housekeeping genes could not be identified or the ST suggested the isolate belonged to another serovar.

### Whole genome assembly

The sequence data were assembled using SPAdes (v.3.13.0) with the careful option and kmers 21, 33, 55 and 77 [56]. The number of contigs, N50 and longest contig in each assembly were identified using QUAST (v.4.6.3) [57]. BWA (v.0.7.12) and SAMtools (v.1.5) were used to determine the percentages of paired and mapped reads and coverage [58, 59]. Isolates with poor quality assemblies were excluded. Prokka v.1.13.3 was used to annotate the assemblies with the rfam option and *Salmonella* specified as the genus [60].

### Phylogenetic analysis

A South African *S*. Infantis reference genome (GCA_922393895.1) was chosen from these isolates with the highest N50 and lowest number of contigs, which was quantified using QUAST v.4.6.3 [57]. PHASTER was used to screen for prophages in the reference; no complete prophages were identified [61]. Snippy v.4.6.0 was used to map and variant call the sequences against the reference using minfrac 0.9 and mapqual 30 [62]. One eBG297 isolate that was extremely divergent from the eBG297 reference (BioSample accession ERS9226551) was excluded. Snippy was also used to create a whole genome alignment which was passed to Gubbins v.2.4.1 to identify putative recombination [63]. Recombination was masked in the generation of a core single nucleotide polymorphism (SNP) alignment by Snippy. A maximum-likelihood phylogeny was generated with RAxML v8.2.12 which was visualized using iToL and rooted to the eBG8 branch [64, 65]. Pairwise-distance matrices were created with mega7 [66].

### AMR, plasmid and virulence factor identification

The AMR, plasmid and virulence factor profiles of the trimmed sequence reads were determined using ARIBA v.2.10.1 with the ResFinder, PlasmidFinder and core vfdb databases, downloaded on 17 January 2022 [67–70]. Mutations associated with causing resistance in the quinolone resistance determining regions of *gyrA*, *gyrB*, *parC* and *parE* were investigated with ARIBA using a database of the wildtype genes from the reference sequence for *S*. Typhimurium LT2 [67]. MDR was defined as resistance to three or more classes of antimicrobials. Phandango was used to generate a heatmap of the results [71].

To assess the presence of pESI, a pseudomolecule was created with an eBG31 reference genome (CP070301) and the pESI contigs from an Israeli human *S*. Infantis whole genome assembly (ASRF01000099–ASRF01000108). Smalt v.0.7.6 (seed=5) was used to map our sequences against this pESI pseudomolecule and SAMtools v.1.5 was used to determine coverage [59, 72]. A heatmap of mapped sequence read coverage was generated in R v.3.5.1 using the packages data.table v.1.11.8, ape v.5.3 and phytools v.0.6 [73–76]. Brig v.0.95 was used with blast v.2.12.0 to compare the pESI-positive isolate with ASRF01000099–ASRF01000108, CP070303.1 and CP016407 [77, 78].

### Genome-wide association study

Pan genome analyses of all *S*. Infantis isolates were performed with Roary v.3.13.0 [79]. Scoary v.1.6.16 was used with the gene presence and absence Roary output, a trait file containing eBG and the phylogeny to calculate associations between the genes and eBG [80]. The results were corrected for multiple comparisons, with a Benjamini–Hochberg adjusted *P*-value of less than 0.05 deemed significant. Venn diagrams were drawn using VennDiagram v.1.6.20 in R [73, 81].

### Recombination and prophage comparison

The number of bases of recombination in the eBG31 isolates were collated from the whole genome Gubbins output [63]. The eBG297 assembly with the largest N50 and smallest number of contigs (GCA_922393915.1) was used as a reference in Snippy with the parameters described above to generate an alignment. Gubbins was used with this alignment to calculate the bases of recombination in the eBG297 isolates relative to their own reference. Box plots were created using R and the Mann–Whitney U test was used to determine significance [73].

The recombination events unique to eBG297 isolates were extracted from the Gubbins output for the whole phylogeny. Each block was nucleotide blasted (v.2.9.0+) against the NCBI nucleotide database; results with less than 90% query coverage were removed and when sorted by E-value the top hit was identified [77].

Prophages were searched for in the *S*. Infantis genomes using PhiSpy (v.4.2.19) [82]. Each prophage found was blasted (v.2.12.0) against the NCBI nucleotide database and hits with query coverage <90% and >110% were removed. A prophage was classed as untypeable if all filtered blast hits matched to parts of a *Salmonella* whole genome assembly.

## Results

### Demographics

The number of reported *S*. Infantis isolates circulating in South Africa fluctuated across the study period of 2004–2016 (Fig. S1). A peak of 116 isolates was observed in 2009; these were collected throughout the year and came from seven of South Africa’s nine provinces.

The majority (89 %, 345/387) of the *S*. Infantis strains were isolated from stool (Table S1a). Thirty isolates were associated with causing invasive infection; 7% (*n*=27) of all isolates were from blood and 0.8% (*n*=3) from cerebrospinal fluid. The number of cases of invasive infection each year ranged from zero to seven. Ten isolates were obtained from urine samples, identified between 2007 and 2015.

Fifty per cent of the isolates were collected from children under the age of 5 years, of which 122 were from infants younger than 1 year. A total of 30 samples were from people over the age of 60 years. An equal proportion of isolates were from males and females. The South African isolate (ERR6753008) included from outside the study period was a female’s blood sample; the UKHSA isolate (SRR1645903) associated with travel to South Africa was from a female’s stool sample.

### Population structure

The population structure of isolates from infections acquired in South Africa was determined from whole genome sequencing. Whilst eBG31 was the dominant eBG (*n*=263, 68 %), 32% (*n*=124) of *S*. Infantis isolates from South Africa belonged to eBG297 (Table S1b). In comparison, 98.9% (*n*=4,837) of the clinical *S*. Infantis isolates in Enterobase were eBG31 and 1.1% (*n*=56) eBG297. All the eBG31 isolates from South Africa were ST32 whereas the eBG297 isolates were split into ST603 (102/124, 82 %), ST1823 (2/124, 2 %), ST7731 (8/124, 6 %) and ST7732 (12/124, 10 %). Both ST7731 and ST7732 were novel to this study and were collected at several time points. Isolates from eBG31 were collected throughout the study period with an increased number of cases in 2009 (Fig. S1); eBG297 strains were also isolated every year. There was no significant association between invasiveness and eBG (*P*=0.16758) as 6.5% (17/263) and 10.5% (13/124) of eBG31 and eBG297 isolates respectively were invasive. The three strains isolated from cerebrospinal fluid (CSF) all belonged to eBG297. A comparable proportion of the isolates from each eBG were from urine at 2.7% (7/263) and 2.4% (3/124) from eBG31 and eBG297 respectively.

**Fig. 1.**
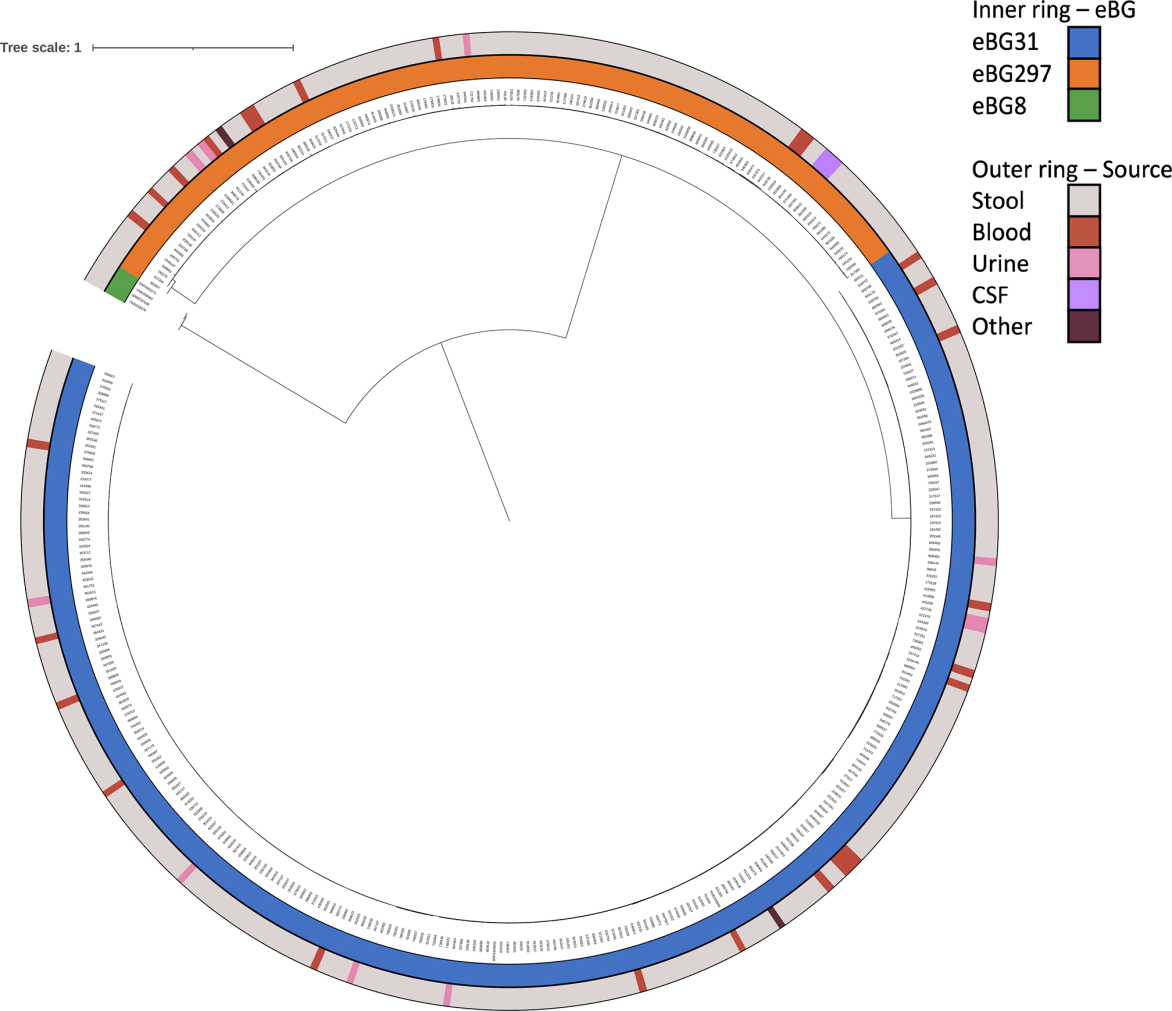
Phylogenetic relationship of eBGs in South Africa. Inner ring, eBG: eBG31 (*n*=262), eBG297 (*n*=124), eBG8 (*n*=4). Outer ring, source: stool (*n*=348), blood (*n*=27), urine (*n*=10), CSF (*n*=3), other (*n*=2). Maximum-likelihood core SNP phylogeny of 386 *S*. Infantis isolates. Isolates belonging to eBG297 comprised 32 % (124/387) of the *S*. Infantis sequences. The median pairwise SNP distance between the eBG31 and eBG297 sequences was 1086. The distance between eBG8 and the *S*. Infantis sequences was four times greater, at 4212 with eBG31 and 4399 with eBG297.

A phylogeny of the *S*. Infantis isolates, and an outgroup of four eBG8 isolates from South Africa, was generated to visualise how closely related eBG31 and eBG297 were (Fig. 1). A cladogram was also created to show the structure within the phylogeny (Fig. S2). As expected, the isolates from each eBG clustered together, each forming a monophyletic clade. The median pairwise SNP distances within each eBG were 12 (range 0–49), 33 (range 0–200) and 73.5 (range 13–79) for eBG31, eBG297 and eBG8 respectively. The eBG31 and eBG297 isolates shared a more recent common ancestor than the eBG8 isolates with a median pairwise SNP distance of 1086 (range 975–1116). eBG31 and eBG297 had a comparable distance to eBG8 at 4212 (range 4202–4236) and 4399 (4365-4413) respectively. The UKHSA travel-associated isolate from 2012 (SRR1645903) was closely related to the other eBG31 isolates; its closest neighbour was one SNP away, indicating that this strain was probably acquired in South Africa (Fig. S2). The majority of the eBG31 isolates from 2009 (88/104) belonged to a single clade (Clade 1, Fig. S2) which also contained 11 isolates from 2010, six from 2011 and six from 2013. The phylogeny was also annotated with source of isolation; minimal clustering of *S*. Infantis from blood or urine was observed as the isolates were distributed throughout the phylogeny (Fig. 1). The strains from CSF clustered together, with a median pairwise SNP distance of zero or one.

**Fig. 2.**
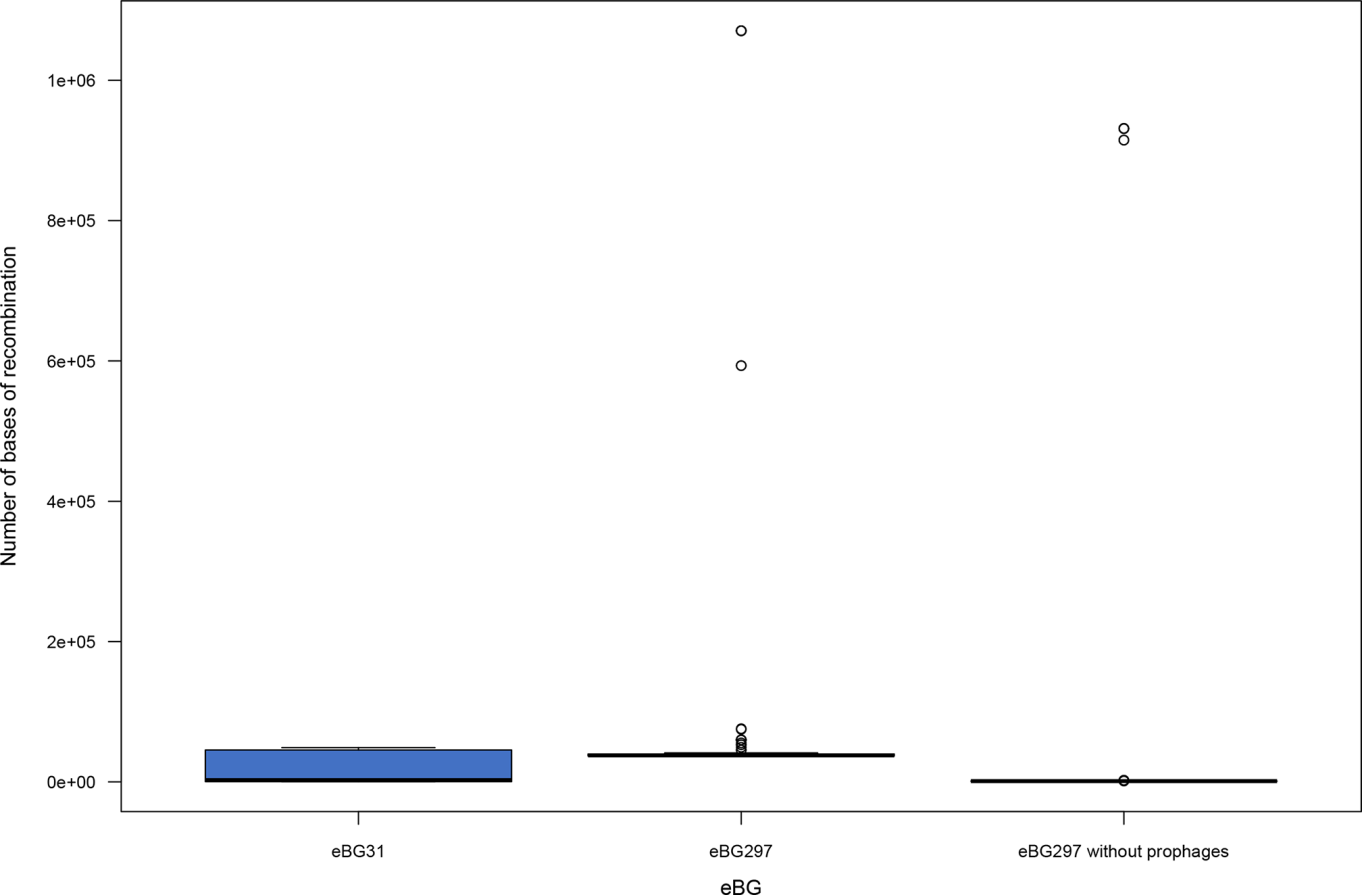
Distribution in the amount of recombination between eBGs in South Africa. Box and whisker plot of the number of bases of recombination in isolates from eBG31 (*n*=263), eBG297 (*n*=124) and eBG297 with prophages masked. Significantly more recombination was present in eBG297 isolates compared to eBG31 (*P*<0.003). This can be attributed to prophages in the eBG297 isolates as masking them significantly decreased the amount of recombination (*P*<2.2×10^−16^).

The extent of recombination in both *S*. Infantis eBGs was compared to determine whether there was an association between eBG and recombination. Fig. 2 illustrates that significantly more recombination was identified in the eBG297 isolates (*P*<0.003). In total, 19% (*n*=1889) of this recombination was predicted to have originated from other *S*. Infantis strains, 10% (*n*=1025) from *S*. Virchow and 5% (*n*=489) from *S*. Heidelberg. When the prophages in the eBG297 reference were masked the amount of recombination significantly decreased (*P*<2.2×10^−16^).

Prophages were present in the majority, 73.4% (91/124), of the eBG297 isolates (Table S1c). Conversely, just 17.5% (46/263) of the eBG31 isolates contained a prophage. *Myoviridae* species were the most common phage in both eBGs. *Salmonella* phage SEN1 was also common in eBG297, present in 27 isolates; however, it was only found in one eBG31 isolate. Two phages were found in just eBG297 isolates and eight were exclusive to eBG31.

### AMR determinants

**Fig. 3.**
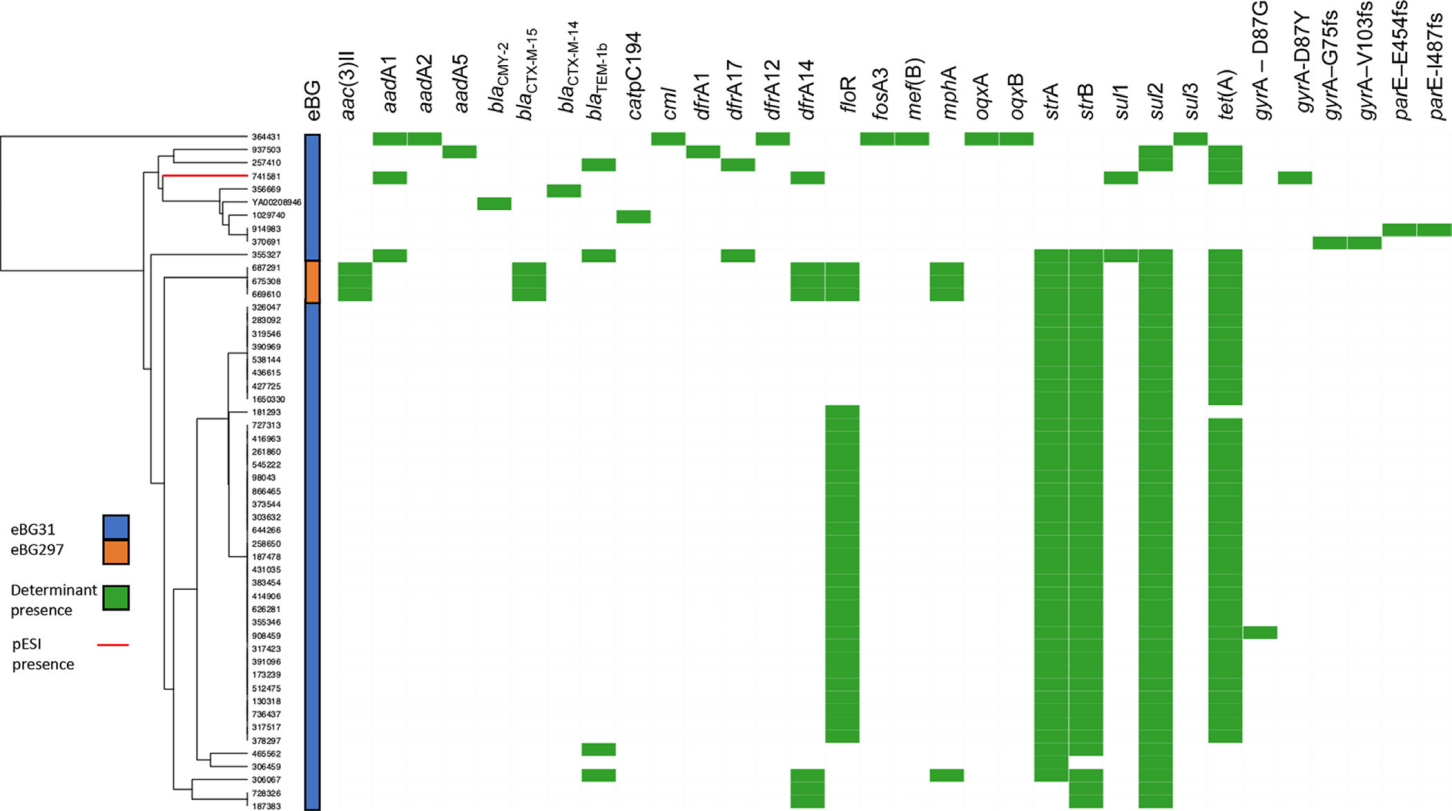
Resistance profile of *S*. Infantis isolates containing AMR determinants. UPGMA dendrogram based on the distance matrix of AMR determinant presence/absence and heatmap of the 49 eBG31 and three eBG297 isolates that contained at least one AMR determinant.

The presence of AMR genetic determinants in the *S*. Infantis isolates was investigated to identify any association with AMR and eBG (Table S1d, e). In total, 86.6% (335/387) of the isolates did not contain any known antimicrobial determinants. Differences between AMR determinants were observed between eBG31 and eBG297 isolates with 15.6% (41/263) of eBG31 and 2.4% (3/124) of eBG297 having MDR. Fig. 3 shows the AMR genes present in each of the 52 isolates that contained at least one AMR determinant and clusters isolates based on AMR determinant presence or absence [67]. The eBG297 isolates with MDR have the same resistance profile; this profile was not observed in the eBG31 isolates. A combination of *strA*, *strB*, *sul*2 and *tet*(A) was present in 37 isolates, conferring resistance to aminoglycosides, sulphonamides and tetracycline, with 28 also resistant to florfenicol due to *floR2* presence. Genes conferring resistance to extended-spectrum β-lactams were present in three eBG297 isolates and one eBG31 isolate.

The majority, 80.4% (311/387), of the *S*. Infantis isolates did not harbour plasmids (Table S1f). pESI was identified in a single isolate (741581), an eBG31 strain isolated from a stool sample in 2013. This isolate had the AMR genes *aadA*1, *sul*1, *dfrA*14 and *tet*(A) and its plasmid was more similar to ASRF01000099–ASRF01000108 than CP070303.1 or CP016407 (Fig. S3). IncA/C plasmids, as determined by the presence of plasmid replicons, were present in 35 of the eBG31 isolates and three eBG297 isolates, which were the MDR isolates described above. Eleven eBG31 isolates and another three eBG297 isolates contained an IncF plasmid and a further three eBG297 and 17 eBG31 isolates contained an IncI plasmid.

### Genome-wide association study

**Fig. 4.**
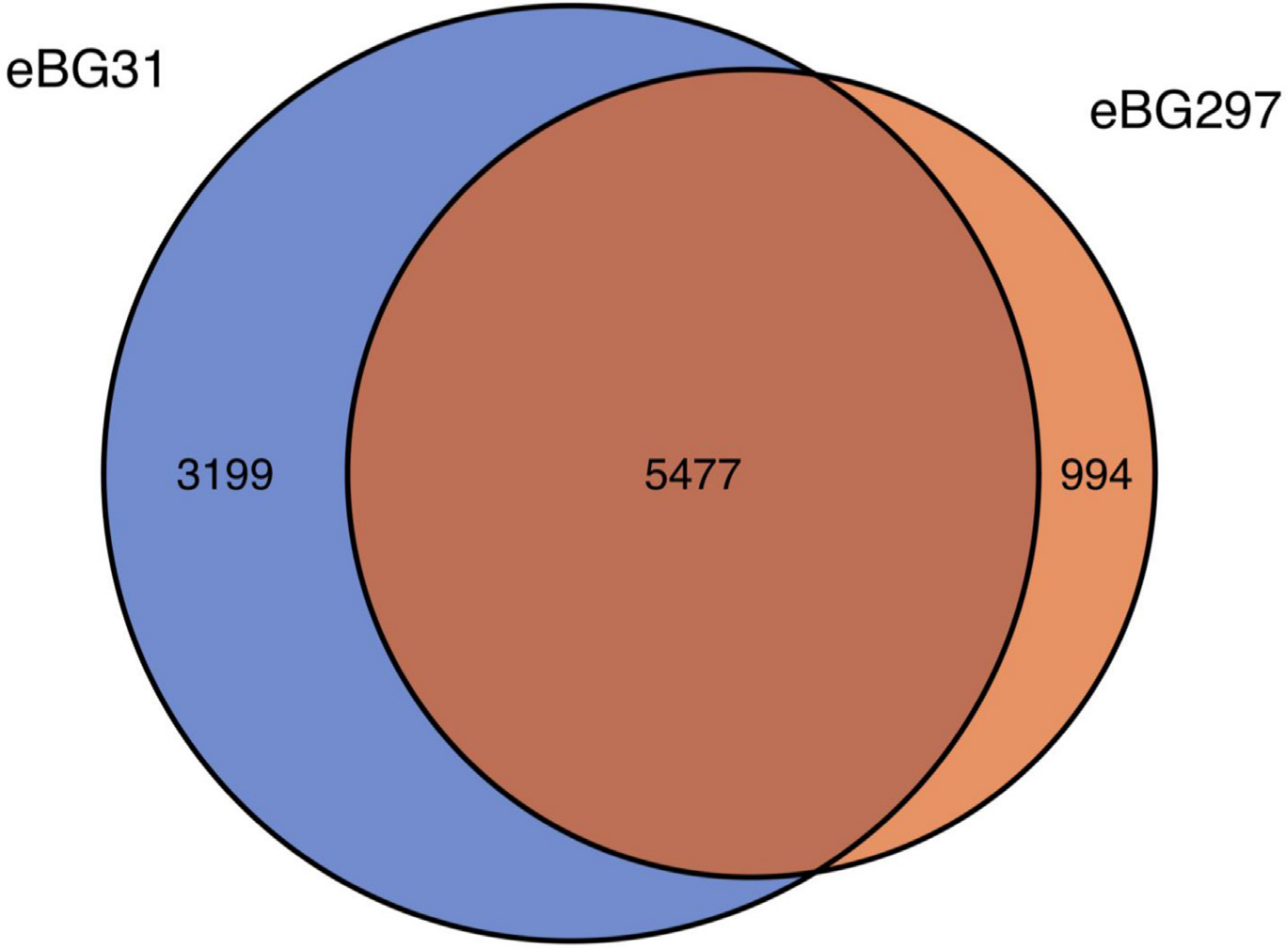
Comparison of the pangenome between eBG31 and eBG297. Venn diagram of the number of genes shared between and unique to 263 eBG31 isolates and 124 eBG297 isolates.

The pan-genomes of isolates from each eBG were compared to further investigate whether the core genome differed between the eBGs. A total of 9670 genes were identified in the *S*. Infantis isolates; 3983 were core genes (present in ≥99% of the isolates) and 5105 were present in than less than 15% of the isolates, showing the diversity in these strains. Fig. 4 shows the number of genes shared between and unique to eBG31 and eBG297. In total, 943 genes were found to be significantly associated with an eBG (*P*<0.05), the majority being found in low numbers of isolates. However, 43 genes were found exclusively in all eBG31 isolates and 34 uniquely in all eBG297 isolates. Examples of the eBG31 exclusive genes include the secreted effector protein *PipB2*, the putative fimbrial-like protein *YfcP* and endoribonuclease *SymE* (Table S2a). Some of the eBG297 exclusive genes were putative fimbrial-like proteins *YadM*, *YadK* and *YfcP*, prophage integrase *IntA* and *SseB* the secreted effector protein.

The virulence factors present in either eBG were compared (Table S1g). Several were present in all the *S*. Infantis isolates such as *sopE2, invH* and *spaR*. Fifteen virulence factors were found in greater than 90% of the eBG31 isolates and a quarter of the eBG297 isolates, including STM0272, STM0274 and *tlde1*. One virulence factor was found in all eBG31 isolates but none of the eBG297 isolates, *lpfD*. The only factor exclusive to eBG297 was sspH1, which was present in 48% of the isolates.

## Discussion

The population structure of South African *S*. Infantis differs from *S*. Infantis isolated elsewhere globally. Whilst just 1.1% (*n*=56) of isolates on Enterobase (accessed 2 February 2022) belonged to eBG297, this eBG comprised 32% (124/387) of the South African *S*. Infantis isolates. Multiple STs were identified in the eBG297 isolates, including two novel ones, ST7731 and ST7732. The increased proportion of eBG297 isolates in South Africa suggests that that eBG may have originated there or, if imported, has found a niche in which to expand. *S*. Infantis isolates from LMICs are poorly represented in both Enterobase and the literature, this being the first detailed analysis on *S*. Infantis from Africa; it is therefore possible that this distribution of eBGs could be observed elsewhere such as other Sub-Saharan African countries. This study has highlighted the importance of local studies where routine sequencing and/or public uploading is not implemented and helps our understanding of diversity and AMR risks within the population. It also emphasizes the benefits of whole genome sequencing, which has enabled investigation of the population structure, AMR and plasmid profiles of *S*. Infantis from an African country for the first time. Further analysis with isolates from food would be beneficial to the study of transmission.

Minimal clustering of blood isolates was present on the phylogeny, indicating that if genetic traits within strains are the cause of invasive *S*. Infantis infection they are not present in the core genome. Calculation of median pairwise SNP distances across the phylogeny demonstrated small distances within eBG31 and eBG297 which could indicate that outbreaks play an important role in the spread of *S*. Infantis in South Africa, facilitated by the burden of HIV [17, 83, 84]; as, for example, *S*. Enteritidis accumulates approximately 1.01 SNPs per genome per year, it may be expected that strains that were not part of an outbreak have a greater SNP distance [85, 86]. The small distance within the eBGs could also indicate that outbreak-associated strains are more likely to be captured and included in the NICD collection.

Comparison with eBG8 illustrated that whilst the median pairwise SNP distance within the eBGs was less than between eBG31 and eBG297, eBG8 was approximately four times as distant from the *S*. Infantis eBGs. This suggests that although eBG297 is genetically distant from eBG31 and present in separate monophyletic clades, it is still worthwhile viewing the isolates as a whole for the purpose of classification. It has previously been speculated that strains which serotype as *S*. Infantis but do not belong to eBG31 occur due to the recombination observed in *Salmonella* [30, 87]. The findings in this research support that, with 73% of the eBG297 isolates containing at least one prophage and significantly more recombination identified in the eBG297 isolates than in eBG31. This increase in recombination can be attributed to prophages as once these were removed from the eBG297 genomes, the extent of recombination decreased significantly.

The NICD reported that the number of human *S*. Infantis isolates recovered in 2009 increased by around 10-fold compared to numbers from previous years (Fig. S1). These numbers started increasing towards the end of April 2009. This coincided with the identification of *S*. Infantis isolated from samples of peanut butter. PFGE analysis of isolates from peanut butter showed PFGE patterns that were indistinguishable from human isolates recovered over the same period, suggestive of relatedness of isolates and that an outbreak was ongoing. However, no formal outbreak investigation was conducted, and so no further information was available or reported (A. M. Smith, personal communication). Now, with whole genome sequence data describing a clade of 111 eBG31 isolates (Clade 1, Fig. S2), the majority isolated in 2009, this is strong evidence that an outbreak did indeed occur. The persistence of strains in this clade into 2013 could indicate that this strain became endemic in South Africa.

A larger proportion of eBG31 isolates (15.6 %, 41/263) contained AMR determinants than in eBG297 (2 %, 3/124), suggesting that eBG297 isolates may occupy a different environmental niche to eBG31 strains. The eBG31 isolates with AMR determinants were isolated throughout the study period, with an increase in 2009, and this equated to just 15% (16/105) of eBG31 isolates from that year, suggesting that the outbreak that year was not associated with AMR. The eBG297 isolates with predicted MDR were all isolated in 2012 from CSF and, alongside 34 eBG31 isolates, contained *strA*, *strB*, *sul*2 and *tet*(A), conferring resistance to aminoglycosides, sulphonamides and tetracycline. A further five AMR genes were present in the eBG297 isolates, suggesting that the eBG297 isolates had ancestors with similar AMR requirements to the eBG31 isolates before occupying a different niche and acquiring these additional genes, or that a plasmid containing the shared AMR genes has spread between the eBGs.

A small proportion (11 %, 44/387) of *S*. Infantis from South Africa contained genes conferring MDR. This is in marked contrast to the levels of AMR seen in *S*. Infantis globally; in 2016 in Europe, 75.3% (496/659) of *S*. Infantis from broiler flocks had MDR [37]. Also, 99.6% (238/239) of strains isolated from humans, poultry farms and chicken carcasses in Ecuador between 2017 and 2018 had MDR [88]. This difference in antimicrobial susceptibility could be attributed to the absence of plasmids in the majority of isolates. pESI has been identified in *S*. Infantis from multiple countries and has been associated with high levels of AMR [7, 8, 40, 41]. However, only one pESI-positive isolate was identified in this study. This plasmid was more homologous to pESI from Israel (ASRF01000099–ASRF01000108) than pESI found in Europe (CP070303.1) or the USA (CP070303.1). It also had the same AMR profile as the Israeli pESI, lacking extended-spectrum beta lactamases such as *bla*_CTX-M-65_ that have been identified in the UK and USA [38, 40, 43]. That pESI has not spread in *S*. Infantis isolates in South Africa could indicate that it does not confer a selective advantage in this location, or has not yet been introduced. However, the prevalence and influence on AMR levels of a pESI-like plasmid in *S*. Infantis elsewhere can be used to infer what would happen if this were to spread in South Africa [38]. Due to our suspected association of *S*. Infantis with outbreaks in South Africa and the high number of immunocompromised individuals, where 13% of the population have HIV [17], any spread of pESI-like plasmids in South Africa has serious implications for public health.

The *S*. Infantis pan-genome showed remarkable diversity, with over half of genes present in less than 15% of the collection. Multiple virulence factors were identified in over 90% of the eBG31 isolates and just a quarter of the eBG297 isolates. These included a *ClpV1* family type VI secretion system (T6SS) ATPase, an *EvpB* family type VI secretion protein and *tlde1*, a T6SS antibacterial effector which is toxic when in the target cell periplasm [89]. *LpfD*, which encodes the tip adhesin of the long polar fimbrial protein [90], was present in all eBG31 isolates but no eBG297 isolates. The presence of these virulence factors could explain the increased prevalence of eBG31 relative to eBG297. Also, a greater proportion of eBG31 isolates had MDR than eBG297. As MDR has been observed in eBG31 in multiple countries including Israel, Switzerland and Italy [7, 8, 40], the higher levels of AMR in eBG31 were expected; that similar levels are not seen in eBG297 suggests that the two eBGs occupy different environmental niches.

To conclude, low levels of AMR determinants were identified in this study, which could suggest that *S*. Infantis endemic in South Africa was not imported from other regions where it is associated with AMR. The population structure of *S*. Infantis isolated in South Africa also differs from that observed elsewhere; analysis of *S*. Infantis from other Sub-Saharan African countries would be beneficial in determining whether this population structure is localized to South Africa.

## Supplementary Data

Supplementary material 1

Supplementary material 2

Supplementary material 3
