# Supplementary material for: Genetic characterization of Salmonella Infantis from South Africa, 2004–2016
Source: Access Microbiol. 2022 Jul 5;4(7):acmi000371. doi: 10.1099/acmi.0.000371 (PMC9394735; doi:10.1099/acmi.0.000371)
Supplement: Supplementary material 3 [file acmi-4-371-s001.pdf]

Supplementary Table and Figure Legends

Table S1. Metadata, MLST type, AMR genes, gyrase mutations and plasmid presence.

a) Metadata for the eBG31, eBG297 and eBG8 isolates included in this study
b) 7-gene MLST type of the *S*. Infantis isolates
c) Prophage presence in the *S*. Infantis isolates
d) AMR gene presence in the *S*. Infantis isolates
e) Mutations in the QRDRs of *gyrA*, *gyrB*, *parC* and *parE* in the *S*. Infantis isolates
f) Plasmid presence in the *S*. Infantis isolates
g) Virulence factors present in the *S*. Infantis isolates

Figure S1. Isolation year of South African *S*. Infantis strains.
Number of *S*. Infantis isolates collected from South Africa between 2004-2016 that were eBG31 and eBG297.

Figure S2. Maximum likelihood core SNP cladogram of eBG31, eBG29 and eBG8 (*Salmonella* Muenchen control). The branches belonging to clade 1 are highlighted in light blue and the UKHSA travel isolate is coloured yellow.
Inner ring, eBG: eBG31 (n=262), eBG297 (n=124), eBG8 (n=4)
Middle ring, source: Stool (n=348), Blood (n=27), Urine (n=10), CSF (n=3), Other (n=2)
Outer ring – Isolated in 2009

Figure S3. Brig plot showing the similarity with the pESI positive isolate 741581 and other pESI plasmids.

a) Comparison with ASRF01000099-ASRF01000108 from Israel
b) Comparison with CP070303.1 from the UK
c) Comparison with CP016407 from the USA

Table S2. Genes found exclusively in an eBG.

a) Genes identified in every eBG31 isolate and zero eBG297 isolates (n=43)
b) Genes identified in every eBG297 isolate and zero eBG31 isolates (n=34)
c) Genes identified in every eBG31 and eBG297 isolate (n=3864)

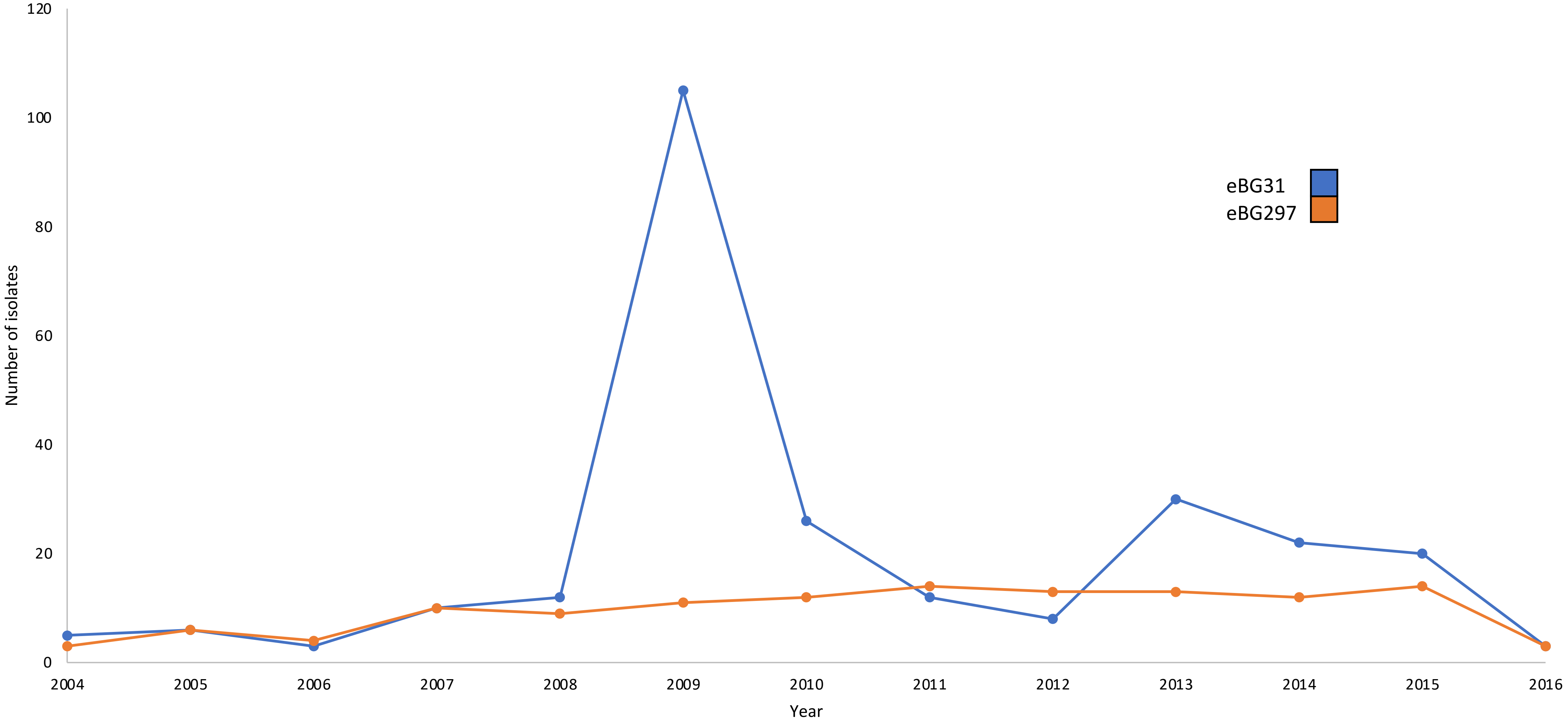

120
100
80
60
40
20
0
Number of isolates
2004
2005
2006
2007
2008
2009
2010
2011
2012
2013
2014
2015
2016
Year
eBG31
eBG297

Figure S1

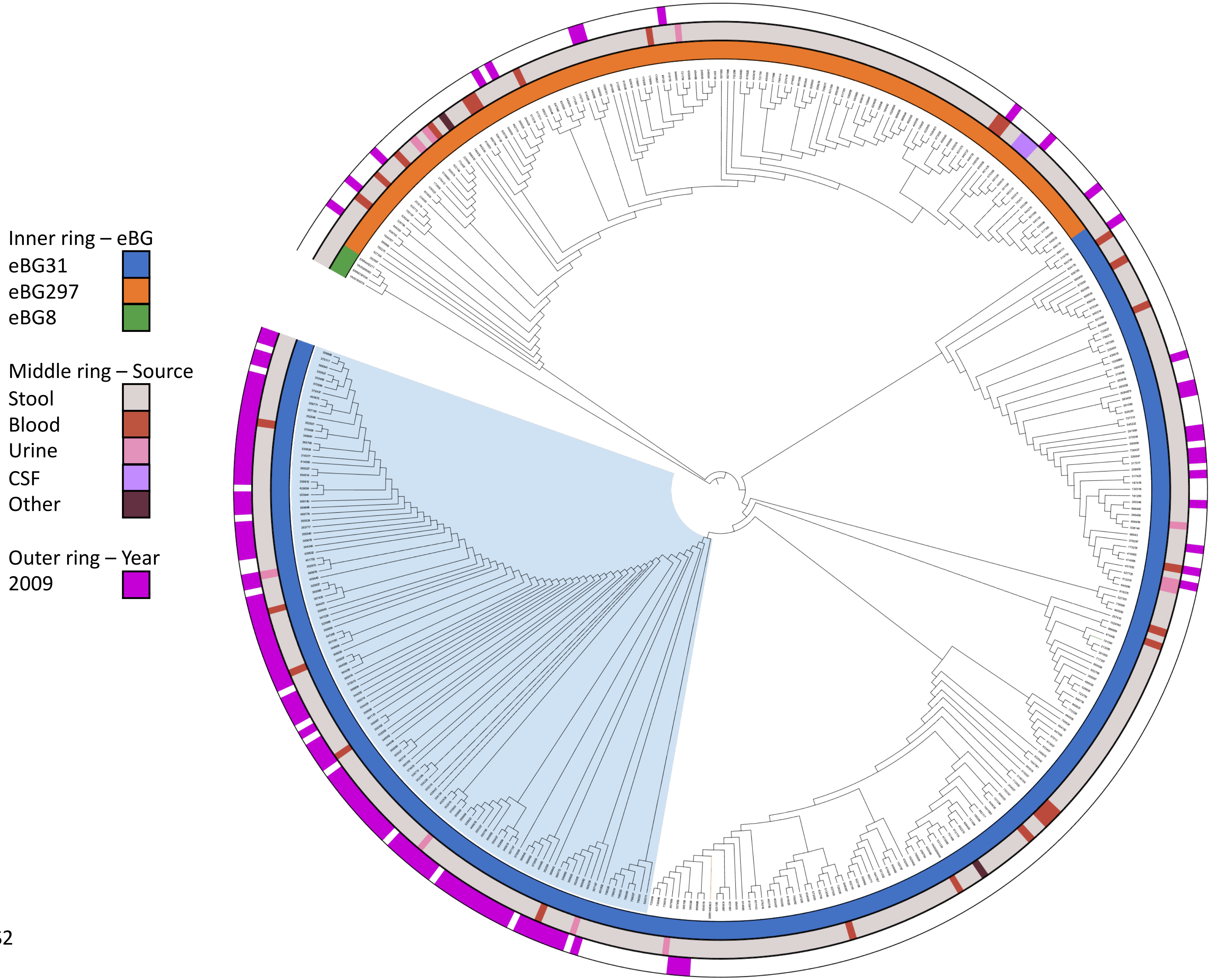
Inner ring – eBG
eBG31
eBG297
eBG8
Middle ring – Source
Stool
Blood
Urine
CSF
Other
Outer ring – Year
2009

Figure S2

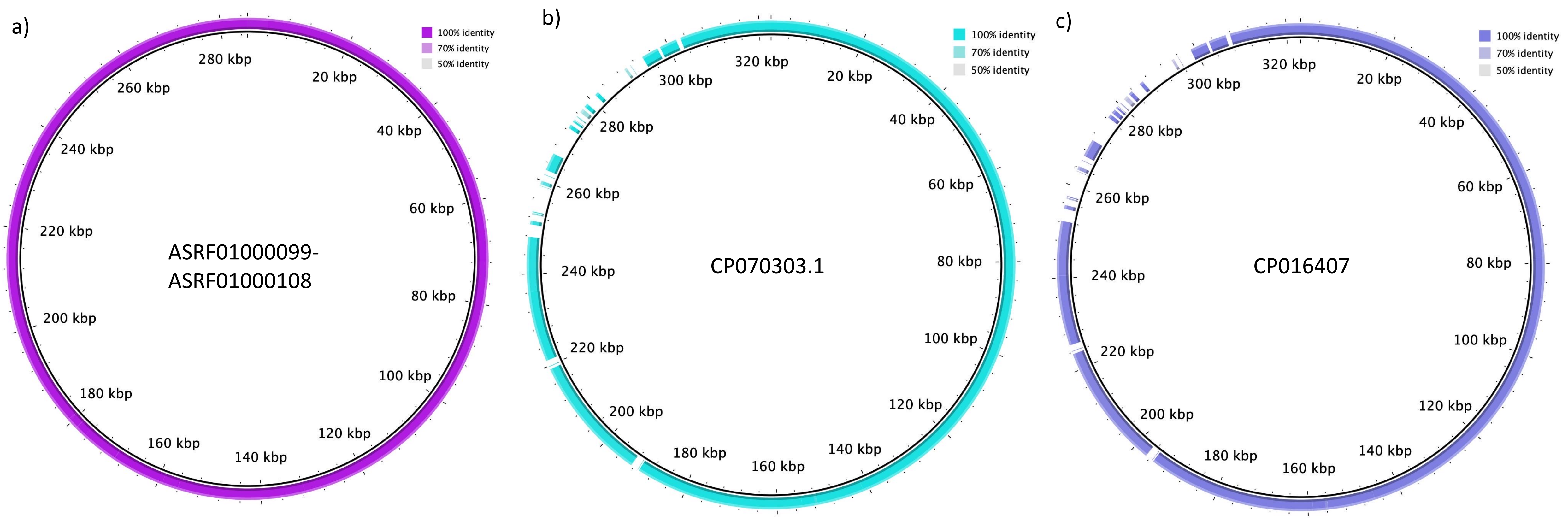
a)
100% identity
70% identity
50% identity
280 kbp
20 kbp
260 kbp
40 kbp
240 kbp
60 kbp
220 kbp
ASRF01000099-
ASRF01000108
80 kbp
200 kbp
100 kbp
180 kbp
160 kbp
140 kbp
120 kbp
b)
100% identity
70% identity
50% identity
320 kbp
20 kbp
300 kbp
40 kbp
280 kbp
60 kbp
260 kbp
240 kbp
CP070303.1
80 kbp
220 kbp
100 kbp
200 kbp
120 kbp
180 kbp
160 kbp
140 kbp
c)
100% identity
70% identity
50% identity
320 kbp
20 kbp
300 kbp
40 kbp
280 kbp
60 kbp
260 kbp
240 kbp
CP016407
80 kbp
220 kbp
100 kbp
200 kbp
120 kbp
180 kbp
160 kbp
140 kbp

Figure S3
